# Comprehensive analysis of the significance of METTL7A gene in the prognosis of lung adenocarcinoma

**DOI:** 10.3389/fonc.2022.1071100

**Published:** 2022-12-23

**Authors:** Ya-Qiang Pan, Ying Xiao, Zhenhua Li, Long Tao, Ge Chen, Jing-Feng Zhu, Lu Lv, Jian-Chao Liu, Jun-Qing Qi, AiZhong Shao

**Affiliations:** ^1^ Department of Cardiothoracic Surgery, Affiliated People’s Hospital of Jiangsu University, Zhenjiang, China; ^2^ Department of Radiation Oncology, The Fourth Affiliated Hospital of Nanjing Medical University, Nanjing, China; ^3^ Department of Thoracic Surgery, Yan’an Affiliated Hospital of Kunming Medical University, Kunming, China

**Keywords:** lung adenocarcinoma, METTL7A, prognosis, cox regression analyses, ceRNA

## Abstract

**Background:**

The most common subtype of lung cancer, called lung adenocarcinoma (LUAD), is also the largest cause of cancer death in the world. The aim of this study was to determine the importance of the METTL7A gene in the prognosis of patients with LUAD.

**Methods:**

This particular study used a total of four different LUAD datasets, namely TCGA-LUAD, GSE32863, GSE31210 and GSE13213. Using RT-qPCR, we were able to determine METTL7A expression levels in clinical samples. Univariate and multivariate Cox regression analyses were used to identify factors with independent effects on prognosis in patients with LUAD, and nomograms were designed to predict survival in these patients. Using gene set variation analysis (GSVA), we investigated differences in enriched pathways between METTL7A high and low expression groups. Microenvironmental cell population counter (MCP-counter) and single-sample gene set enrichment analysis (ssGSEA) methods were used to study immune infiltration in LUAD samples. Using the ESTIMATE technique, we were able to determine the immune score, stromal score, and estimated score for each LUAD patient. A competing endogenous RNA network, also known as ceRNA, was established with the help of the Cytoscape program.

**Results:**

We detected that METTL7A was down-regulated in pan-cancer, including LUAD. The survival study indicates that METTL7A was a protective factor in the prognosis of LUAD. The univariate and multivariate Cox regression analyses revealed that METTL7A was a robust independent prognostic indicator in survival prediction. Through the use of GSVA, several immune-related pathways were shown to be enriched in both the high-expression and low-expression groups of METTL7A. Analysis of the tumor microenvironment revealed that the immune microenvironment of the group with low expression was suppressed, which may be connected to the poor prognosis. To explore the ceRNA regulatory mechanism of METTL7A, we finally constructed a regulatory network containing 1 mRNA, 2 miRNAs, and 5 long non-coding RNAs (lncRNAs).

**Conclusion:**

In conclusion, we presented METTL7A as a potential and promising prognostic indicator of LUAD. This biomarker has the potential to offer us with a comprehensive perspective of the prediction of prognosis and treatment for LUAD patients.

## Introduction

Lung cancer has been the leading cause of cancer-related fatalities for the past many decades, and its incidence has been almost the greatest worldwide (1). Typically, lung cancer is divided into two pathological categories: small cell lung cancer (SCLC) as well as non-small cell lung cancer (NSCLC), lung adenocarcinoma (LUAD) is the most common histological subtype of NSCLC, which accounts for approximately half of all lung cancer (2). Despite continual advances in the technology of LUAD detection and treatment, the death rate associated with this disorder has not dropped considerably (3). Overall survival (OS) remains inadequate due to a deficiency of fresh biological markers connected with the prognosis of LUAD, despite the fact that molecular targeted treatment and immunotherapy have made significant progress. In addition, when advanced LUAD is detected, surgical treatment is not possible because the initial tumor lesion has spread to adjacent tissues or organs (4). Therefore, it is essential to determine the major prognostic signs for LUAD.

In recent years, the field of biology known as epigenetics has experienced significant growth. Its role in the development and evolution of malignancies is becoming more and more obvious as research continues (5, 6). DNA modification, RNA modification, chromatin remodeling, and the translation of histones are the major forms of epigenetic regulation. The regulation of gene expression by epigenetic processes is a process that is both complicated and reversible (7–9). In biological or pathological mechanisms, the changes are essential for processing environmental stimuli and regulating the expression of associated genes (10, 11). As the most widespread messenger RNA (mRNA) modification, N6-methyladenosine (m6A) plays a crucial role in both physiological and pathological processes (12, 13). m6A is a ubiquitous modification of RNA molecules in eukaryotic organisms. Changes in m6A are essential for mRNA splicing, export, translation, and stability (14). Numerous enzymes, which including methylases methyltransferase-like 3 (METTL3) and Heterogeneous Nuclear Ribonucleoprotein A2/B1 (HNRNPA2B1), are involved in the m6A system. In addition to this, there is a significant link between m6A and sickness. Recent studies have shown that alterations to the m6A gene play a part in the progression of a number of adult diseases, including obesity (15) and cancer (16), as well as embryonic development (17). As was mentioned before, a considerable amount of focus has been placed on the role that m6A plays in the clinical diagnosis and therapeutic prediction capabilities of LUAD (18, 19). However, neither the expression of Methyltransferase-like protein 7A (METTL7A) in LUAD nor its prognostic importance has been established.

During the course of this study, the expression pattern of METTL7A mRNA in LUAD has been revealed. The information needed for this investigation was found by searching databases called The Cancer Genome Atlas (TCGA) and Gene Expression Omnibus (GEO). The experimental approach utilizing RT-qPCR showed a pattern consistent with our studies at the expression level. Furthermore, we investigated the association between METTL7A and the clinical characteristics of LUAD patients included in the TCGA database. These variables include age, sex, and pathological stage of the disease. Analysis of LUAD patients with high levels of METTL7A expression showed that these individuals had a greater chance of survival. This work uses transcriptomic data from public databases and analyzes the data with methods from the field of bioinformatics to gain insight into the role and regulatory mechanisms of METTL7A in LUAD prognosis. The results of this study lay a theoretical foundation for clinical prediction and treatment.

## Materials and methods

### Data source

We obtained transcriptomic data for 526 LUAD samples and 59 normal samples using the TCGA database (https://portal.gdc.cancer.gov/). Of these, 493 LUAD patients with complete clinical information were selected for the study of OS and clinical relevance. Information on 447 LUAD patients with disease-free survival (DFS) was selected for DFS analysis. The GSE32863 (20), GSE31210 (21) and GSE13213 (22) datasets are from the GEO database (https://www.ncbi.nlm.nih.gov/geo/). To validate METTL7A expression in paracancer and LUAD samples, GSE32863 dataset was used. The GSE31210 and GSE13213 datasets were used to validate the results of survival analysis.

### Sample sources for RT-qPCR validation

At the People’s Hospital Affiliated to Jiangsu University, we collected surgically resected frozen tumors and paracancerous tissue from 20 patients with pathological diagnosis of LUAD. Immediately after removal, surgical specimens were placed in liquid nitrogen and frozen to -80°C. The Ethics Committee of the People’s Hospital Affiliated to Jiangsu University supervised and approved the research project (approval number K-20220088-W). Both patients and volunteers gave express written consent to participate in this research investigation.

### Identification of the METTL7A expression in pan-cancer

Both the TIMER database (23) (http://timer.cistrome.org/) and the GEPIA database (24) (http://gepia.cancer-pku.cn/) were searched for information on the expression of METTL7A in pan-cancer.

### Survival analysis

LUAD patients were divided into two groups, namely high- and low-expression groups, with the ideal cut-off value of gene expression serving as the threshold. This was done so that the function that METTL7A plays in the survival of patients could be determined. For the purpose of conducting a survival analysis, the ‘survival’ R package (version 3.1.12) was employed, and a Kaplan–Meier curve was produced. When the P-value was less than 0.05, researchers considered there to be a significant difference in survival rates between the high expression and low expression groups.

### Correlation between the METTL7A expression and other clinicopathological characteristics

The Wilcoxon test or the Kruskal-Wallis test was used to assess whether a link existed between the expression of METTL7A and the related clinicopathological features. Gender, age, pathological T, N, M stage, and tumor stage were among these variables. This association was represented using a violin plot. In addition, a K-M survival analysis was performed on the identified subtypes with distinct clinicopathological features.

### Independent prognostic analysis and construction of a nomogram

In the TCGA-LUAD dataset, we performed a univariate cox regression analysis followed by a multivariate Cox regression analysis to identify independent predictors of OS or DFS. After that, the ‘cph’ function in R was used to generate a nomogram that included the independent prognostic variables. The calibration curves and ROC curves for one, three, and five years were constructed in order to further validate the nomogram’s capacity for accurate prediction and efficiency.

### Gene set variation analysis

We used the “GSVA” R package (25) to analyze changes in enriched pathways in high and low expression groups based on Hallmark genes downloaded from MsigDB (http://www.gsea-msigdb.org/gsea/msigdb/index.jsp) set. In addition, we compared the enrichment analysis of the high and low expression groups. The value of |t| must be > 1 for the Hallmark genome to be considered significantly enriched in the pathway. Further calculations were performed to determine differences in enriched pathways in the low and high expression groups, and matching heatmaps are shown.

### Estimation of the tumor microenvironment

The ‘Estimation of Stromal and Immune cells in MAlignant Tumours using Expression data’ (ESTIMATE) method was performed to determine the stromal-, immune- and estimate-scores of each LUAD individual in both the low-expression and high-expression groups (26). The enrich score of immunological infiltration cells was computed using single-sample gene set enrichment analysis (ssGSEA) (27) and the Microenvironment Cell Populations-counter (MCP-counter) method (28). The violin plot was developed so that the changes in immune cell infiltration that occurred between the low-expression and high-expression groups could be shown. As was done in the previous publication (29), the geometric mean of the expression of GZMA and PRF1 in transcripts per million (TPM) was used to compute the cytolytic activity (CYT) of each LUAD patient in the high- and low-expression subgroups.

### Construction of the ceRNA regulatory network

The ‘limma’ package was utilized with the screening criteria of |log2FC|>0.5 and FDR<0.05 in order to identify the differentially expressed microRNAs (DE-miRNAs) and long noncoding RNAs (DE-lncRNAs) between LUAD samples and normal samples. This was the first step in the process. After that, we predicted the interaction between METTL7A and DE-miRNAs by using the miRWalk website with a confidence level of 1, and we only kept the relationship pairings that had opposing expression patterns in accordance with the ceRNA mechanism. In a similar manner, we utilized StarBase to predict the interaction between DE-lncRNAs and DE-miRNAs. However, we only kept the pair combinations that showed opposing expression patterns. Cytoscape (30) was used to design the METTL7A-DElncRNA-DEmiRNA ceRNA network, which was the last step.

### RNA extraction and quantitative real-time polymerase chain reaction

Using the Nuclezol LS RNA Isolation Reagent and according to the protocols provided by the manufacturer (ABP Biosciences Inc.), total RNA was extracted from both the 10 normal tissue samples and the 10 LUAD tissue samples. Next, using the SureScript-First-strand-cDNA-synthesis-kit (GeneCopoeia) and following the instructions provided by the manufacturer, total RNA was converted into cDNA by the process of reverse transcription. qPCR was then carried out with the use of the BlazeTaq™ SYBR ^®^ Green qPCR Mix 2.0 (GeneCopoeia). The following thermocycling conditions were used for quantitative polymerase chain reaction (qPCR): one cycle at 95°C for one minute (initial denaturation), followed by forty cycles of twenty seconds at 95°C (denaturation), twenty seconds at 55°C(annealing), and thirty seconds at 72°C (extension). **Table 1** included a rundown of the primer sequences for your perusal. Calculations were done with the 2−ΔΔCq technique (31) after the relative expression level was first standardized with respect to the endogenous control GAPDH. The student t-test d was utilized to analyze the differences that were found between the two groups. In statistical analysis, a result was considered statistically significant if the two-tailed P-value < 0.05.

**Table 1:** The sequences of primers for Qpcr.

Primer	Sequences	
METTL7A For	GATGGCTCTGTGGATGTGGT	
METTL7A Rev	GCTCTCTCTGGTCAGGTTGC	
GAPDH For	CCCATCACCATCTTCCAGG	
GAPDH Rev	CATCACGCCACAGTTTCCC	

### Statistical analysis

The programming language R was used to carry out all of the studies, and the Wilcoxon test and the Kruskal-Wallis test were utilized in order to compare the data obtained from the various groups. In the event that it was not indicated above, a P-value of less than 0.05 was regarded as statistically significant.

## Results

### METTL7A was down-regulated in pan-cancer including LUAD

**Figure 1:**
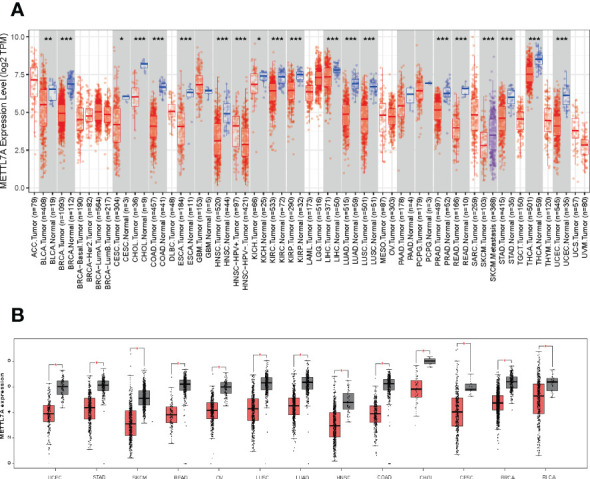
METTL7A gene expression and genetic changes in cancer. **(A)** METTL7A expression profiles in the pan-cancer dataset. **(B)** METTL7A is highly overexpressed in thirteen cancer tissues. *p < 0.05; **p < 0.01; ***p < 0.001. METTL7A, Methyltransferase-like protein 7A.

**Figure 2:**
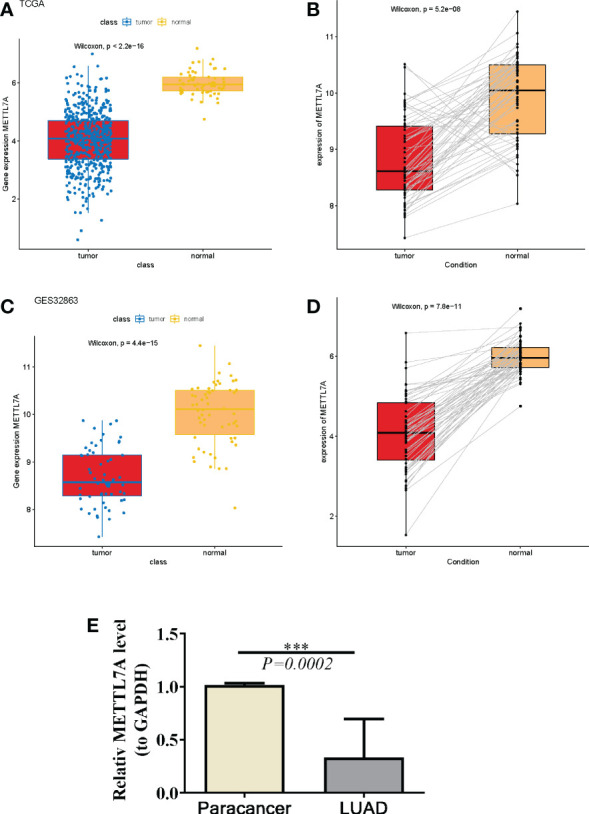
The same expression and prognostic value of METTL7A in LUAD. **(A)** The expression of METTL7A in LUAD tissues and normal tissues based on the TCGA-LUAD database. **(B)** The expression profles of the METTL7A in the paired LUAD and normal samples in the TCGA-LUAD dataset. **(C)** The expression of METTL7A in LUAD tissues and normal tissues based on the GEO database. **(D)** The expression profles of the METTL7A in the paired LUAD and normal samples in the GEO dataset. **(E)** qRT-PCR results of METTL7A in LUAD tumor tissues and adjacent normal tissues. ***p < 0.001. METTL7A, Methyltransferase-like protein 7A; LUAD, lung adenocarcinoma; qRT-PCR, quantitative real-time polymerase chain reaction.

Compared to corresponding normal samples, we detected that METTL7A was down-regulated in18 types of cancer, which include BLCA, BRCA, CESC, CHOL, COAD, ESCA, HNSC, KICH, KIRC, KIRP, LIHC, LUAD, LUSC, PRAD, READ, STAD, THCA, and UCEC, based on the data from TIMER and GEPIA database (**Figures 1A, B**). To further investigate the expression level of METTL7A in LUAD individuals, we examined the expression of METTL7A in normal and LUAD individuals, as well as matched paracancer and LUAD samples from the TCGA-LUAD and GSE32863 datasets. As demonstrated in **Figures 2A-D**, METTL7A was definitely down-regulated in LUAD individuals relative to normal individuals or matched paracancer samples, implying a suppressive effect in LUAD development. To further confirm the expression level of METTL7A in clinical LUAD and paracancer individuals, 10 pairs of clinical samples were obtained, their RNA was extracted, and RT-qPCR was conducted. METTL7A was down-regulated in the LUAD samples, as shown in **Figure 2E**, similar with the results from the public database.

### METTL7A was associated with age and T stage for LUAD

**Figure 3:**
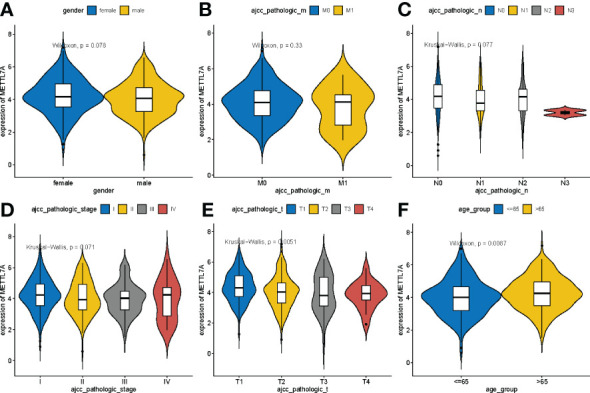
Variations in expression of METTL7A between clinical characteristics. **(A)** Variations in expression of METTL7A based on the gender. **(B)** Variations in expression of METTL7A during the M stage. **(C)** Variations in expression of METTL7A based on N stage. **(D)** Variations in expression of METTL7A based on tumor stage. **(E)** Variations in expression of METTL7A based on the T stage. **(F)** Variations in expression of METTL7A based on age. METTL7A, Methyltransferase-like protein 7A.

To identify the relationship between METTL7A expression level and clinicopathological characteristics, we analyzed the variances in METTL7A expression among subgroups of clinical factors. As shown in **Figure 3**, METTL7A expression was linked with age and T stage (p < 0.05). The expression of METTL7A was higher in patients aged > 65 than in those aged ≤ 65, and the expression of M gradually decreased from the T1 stage to the T3 stage.

### METTL7A was associated with the survival of LUAD patient

**Figure 4:**
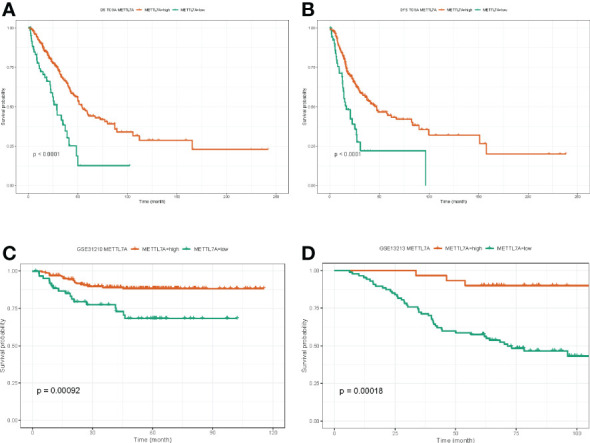
The prognostic value of METTL7A in LUAD. **(A)** The associations between METTL7A expression and overall survival of LUAD patients. **(B)** The associations between METTL7A expression and disease-free survival of LUAD patients. **(C)** The associations between METTL7A expression and overall survival of GEO patients. **(D)** The associations between METTL7A expression and disease-free survival of GEO patients. METTL7A, Methyltransferase-like protein 7A; LUAD, lung adenocarcinoma.

Next, we investigated the role of METTL7A in LUAD prognosis. We discovered that the overall survival (OS) rate and disease-free survival (DFS) rate of the METTL7A high-expression subgroup in the TCGA-LUAD dataset were both substantially greater than those of the METTL7A low-expression subgroup (**Figures 4A, B**). In accordance with the findings from the TCGA-LUAD database, the OS of the METTL7A high-expression subgroup was greater compared to the METTL7A low expression group in both the GSE31210 and GSE13213 datasets (**Figures 4C, D**). The above results suggested that METTL7A was a protective factor in the prognosis of LUAD.

**Figure 5:**
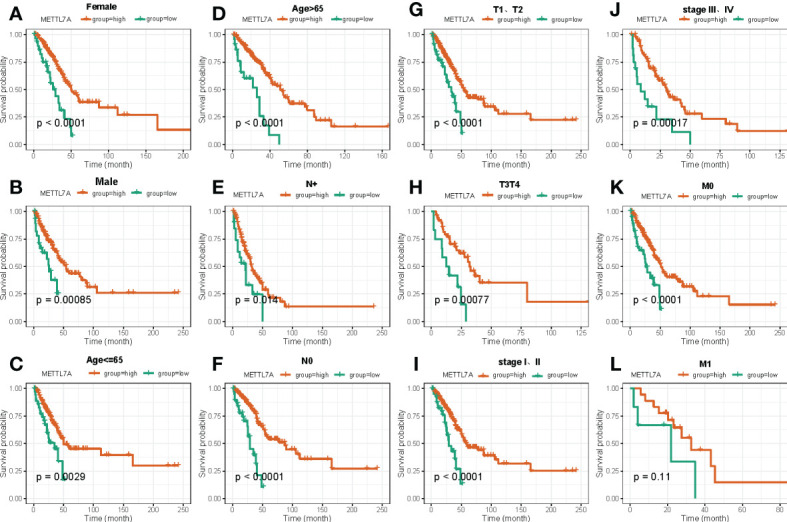
The Kaplan–Meier curve of independent prognostic factors for overall survival between high and low risk groups based on clinical features in LUAD. **(A)** Kaplan-Meier survival curves show differences in survival between high and low risk groups based on Female. **(B)** Kaplan-Meier survival curves show differences in survival between high and low risk groups based on Male. **(C)** Kaplan-Meier survival curves show differences in survival between high and low risk groups based on age ≤ 65. **(D)** Kaplan-Meier survival curves show differences in survival between high and low risk groups based on age>65. **(E)** Kaplan-Meier survival curves show differences in survival between high and low risk groups based on N+ stage. **(F)** Kaplan-Meier survival curves show differences in survival between high and low risk groups based on N0 stage. **(G)** Kaplan-Meier survival curves show differences in survival between high and low risk groups based on T1 and T2 stage. **(H)** Kaplan-Meier survival curves show differences in survival between high and low risk groups based on T3 and T4 stage. **(I)** Kaplan-Meier survival curves show differences in survival between high and low risk groups based on stage I-II. **(J)** Kaplan-Meier survival curves show differences in survival between high and low risk groups based on stage III-IV. **(K)** Kaplan-Meier survival curves show differences in survival between high and low risk groups based on M0 stage. **(L)** Kaplan-Meier survival curves show differences in survival between high and low risk groups based on M1 stage. LUAD, lung adenocarcinoma.

In the following, we performed the survival analysis of LUAD patients under different subgroups of clinical factors, and the result was displayed in **Supplementary Figure S1**. After that, we utilized stratified survival analysis to determine whether or not the METTL7A was connected to the survival of LUAD individuals under various clinicopathological features. As shown in **Figure 5**, we discovered that the level of expression of METTL7A was capable of effectively predict OS for all subgroups based on various clinical features, with the exception of M1 stage.

### Independent prognostic analysis and nomogram construction

**Figure 6:**
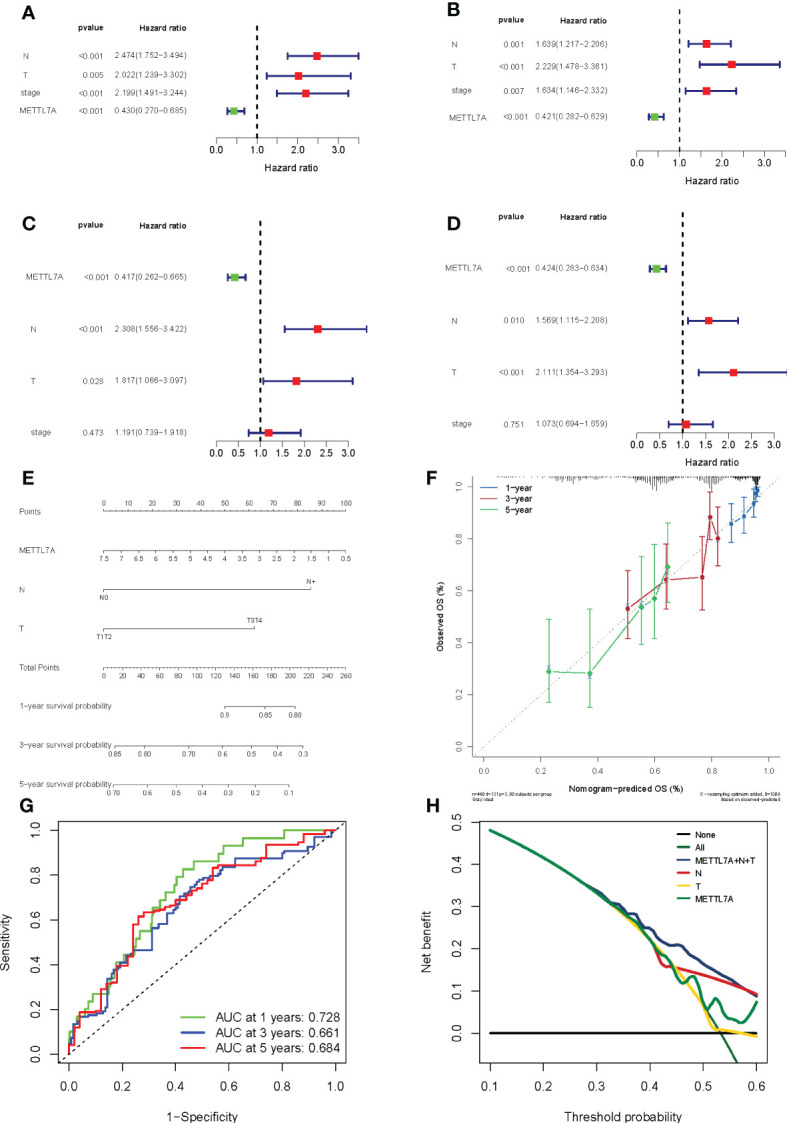
Forest plot for risk factors, nomogram and validation results in LUAD. **(A)** The prognostic risk factors identified using a univariate Cox regression model based on TCGA dataset. **(B)** The prognostic risk factors identified using a multivariable Cox regression model based on TCGA dataset. **(C)** The prognostic risk factors identified using a univariate Cox regression model based on GEO dataset. **(D)** The prognostic risk factors identified using a multivariable Cox regression model based on GEO dataset. **(E)** The performance of the nomogram in facilitating clinicians to predict 3- and 5-year OS. Sum of each score for the risk variables, including risk scores and clinicopathological characters, to predict OS. **(F)** The accuracy of the risk score’s ability to predict patient outcomes at 1, 3, and 5 years was evaluated using ROC curves. **(G)** Area under the curve was calculated on the prognostic model at 1, 3, and 5 years. **(H)** Decision Curve Analysis curve was calculated on the prognostic model. LUAD, lung adenocarcinoma; ROC, receiver operating characteristic; OS, overall survival.

We conducted univariate and multivariate Cox regression analyses in order to further assess whether the expression of METTL7A was independent of those other clinical features (age, gender, pathologic M, pathologic N, pathologic T, and pathologic stage) as a prognostic factor for LUAD individuals. According to the results of the univariate analysis, the pathologic N, pathologic T, pathologic stage, and METTL7A were linked with the patients’ overall survival and disease-free survival (**Figures 6A, B**). According to the findings of the multivariate Cox regression studies, the METTL7A can be utilized independently to predict the overall survival and disease-free survival of LUAD individuals (**Figures 6C, D**). In addition, the multivariate analysis revealed that pathologic N and pathologic T are both independent prognostic variables. Following that, we developed a nomogram to predict the 1-, 3-, and 5-year overall survival of individuals based on independent prognostic markers (**Figure 6E**). According to the calibration plots, the nomogram was effective for predicting the 1-, 3-, and 5-year survival probability in LUAD individuals (**Figures 6F–H**).

### GSVA between the METTL7A high- and low-expression groups

**Figure 7:**
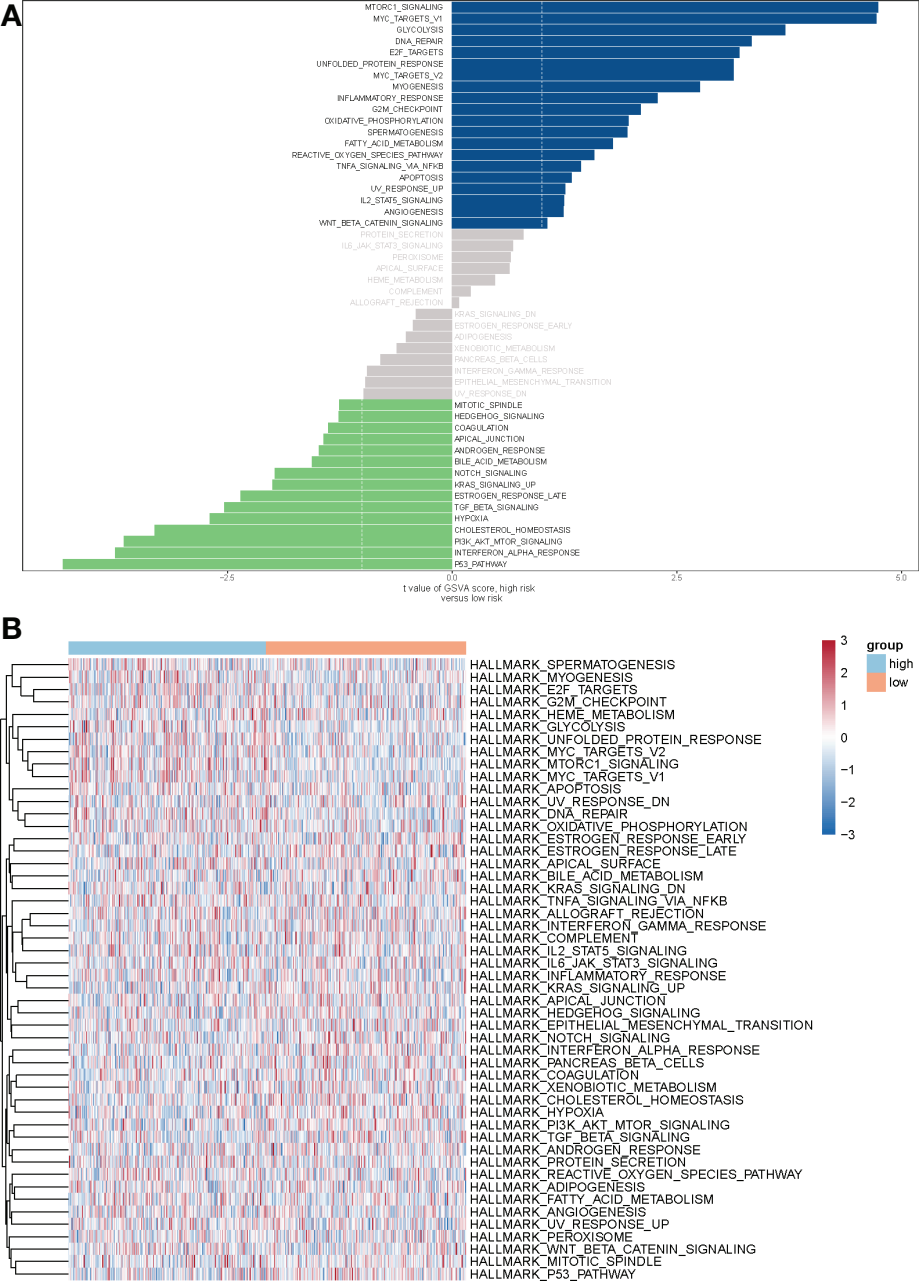
The analysis of GSVA indicates functional differences between high- and low-risk subgroups. **(A)** Variations in pathway activity evaluated by GSVA between patients at high and low risk. The T values are shown using a linear model. The blue column represents active pathways in individuals at high risk, whereas the green column represents activated pathways in patients at low risk. **(B)** The results of the heat map showed that 20 pathways were enriched in the high-expression group and 15 pathways were enriched in the low-expression group. Orange red represents the low-risk group, light blue represents the high-risk group. GSVA, Gene set variation analysis.

To explore the molecular mechanisms underlying the differential prognosis of the METTL7A high- and low-expression groups, we performed a GSVA analysis based on the hallmark gene set. The results showed that 20 pathways were enriched in the high-expression group and 15 pathways were enriched in the low-expression group (**Figures 7A, B**). ‘MTORC1 signaling’, ‘glycolysis’, ‘DNA repair’, ‘inflammatory response’, ‘G2M checkpoint’, ‘oxidative phosphorylation’, ‘TNFA signaling *via* NFKB’, ‘IL2 STAT5 signaling’, and ‘WNT beta catenin signaling’ were enriched in the high-expression group. ‘Mitotic spindle’, ‘HEDGEHOG signaling’, ‘bile acid metabolism’, ‘NOTCH signaling’, ‘KRAS signaling up’, ‘TGF beta signaling’, ‘hypoxia’, ‘cholesterol homeostasis’, ‘PI3K AKT MTOR signaling’, ‘interferon alpha response’, and ‘p53 pathway’ were enriched in the low-expression group.

### The difference of TME between the METTL7A high- and low-expression groups

**Figure 8:**
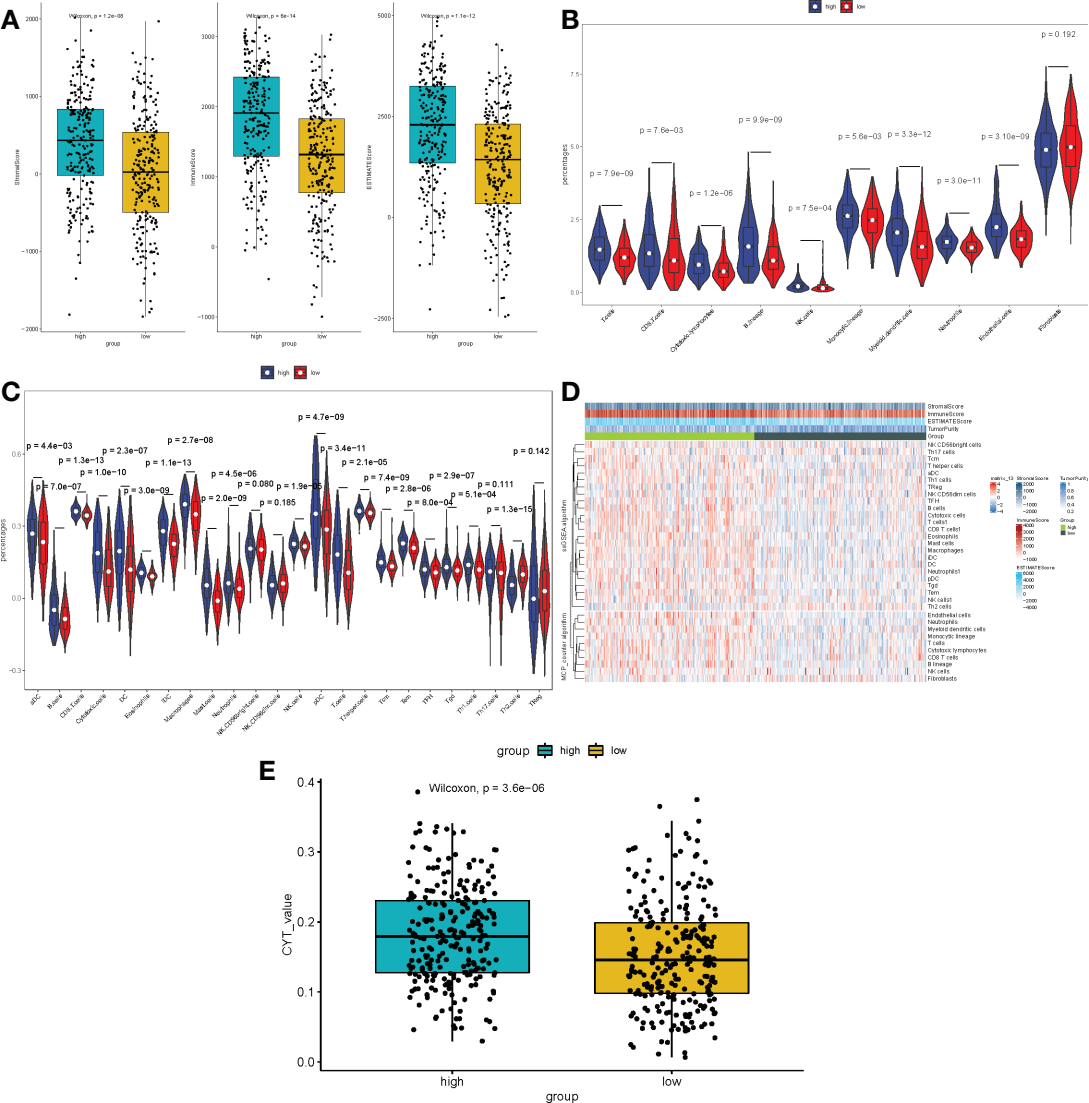
Immune scores and stromal scores correlate with subtypes of LUAD. **(A)** The P values for the distribution of immune scores, stromal scores, and ESTIMATE scores in high and low risk groups, and all are less than 0.05. **(B)** Violin plot displaying the percentage of 10 immune cells and 28 immune cells in the TCGA-LUAD sample utilizing MCP-counter and ssGSEA, respectively. **(C)** The heat plot of various immune cell infiltration levels between high-risk and low-risk patients as determined by ssGSEA. **(D)** The violin plot of the cytolytic activity levels between high-risk and low-risk patients as assessed. LUAD, lung adenocarcinoma; ssGSEA, single-sample gene set enrichment analysis; ESTIMATE, Estimation of Stromal and Immune cells in MAlignant Tumours using Expression data; MCP-counter, Microenvironment Cell Populations-counter. CYT, cytolytic activity. (E) The CYT of each patient with LUAD in the low-expression and high-expression group was estimated.

Because multiple immune-related pathways were significantly enriched in both the low- and high-expression subgroups, and the tumor microenvironment (TME) is an essential component in the development of tumors. Following that, we carried out a comparative study between the METTL7A low-expression and high-expression subgroups by performing a differential analysis of immune-, stromal, and estimate- scores. As displayed in **Figure 8A**, the scores for immune, stromal, and estimation were all considerably higher in the high-expression group than they were in the low-expression group. After that, we performed an analysis on the immune infiltrating cells in both the low-expression and high-expression subgroups of METTL7A. The findings of the MCP-counter indicated that the percentage of all cell types, with the exception of fibroblasts, was higher in the subgroup with higher levels of METTL7A expression than low expression subgroup (**Figures 8B, D**). According to the findings of ssGSEA, the enrich score of high-expression subgroup of aDC, B cells, CD8 T cells, Cytotoxic cells, DC, Eosinophils, iDC, Macrophages, Mast cells, Neutrophils, NK cells, pDC, T cells, T helper cells, Tcm, Tem, TFH, Tgd, and Th1 cells was significantly higher than the enrich score of low-expression subgroup. However, compared to the high-expression group, the low-expression group had a considerably higher enrich score for Th2 cells (**Figures 8C, D**). There was an association between the cytolytic activity and inflammation. In addition, the CYT of each patient with LUAD in the low-expression and high-expression group was estimated and exhibited in **Figure 8E**. Our findings also showed that the CYT of the high-expression subgroup was considerably greater than the low-expression subgroup. The above data revealed that the immunological microenvironment of the low-expression subgroup was suppressed, which may be connected to the poor prognosis.

### ceRNA regulatory network of METTL7A

**Figure 9:**
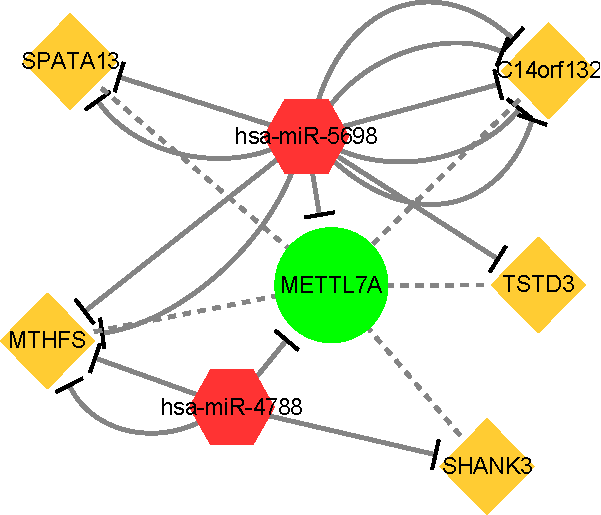
The ceRNA regulation network of 5 lncRNAs, 2 miRNAs, and 1 mRNA in patients. The yellow rectangle indicates lncRNAs; the red diamond indicates miRNAs; the green circle indicates mRNA. lncRNA, long non-coding RNA; miRNA, microRNA.

To explore the ceRNA regulatory mechanism of METTL7A, we finally constructed a regulatory network containing 1 mRNA, 2 miRNAs, and 5 lncRNAs as described in Materials and Methods 2.9 (**Figure 9**). The hsa-miR-5698 and hsa-miR-4788 regulated the expression of METTL7A. C14orf132, SPATA13, MTHFS, and TSTD3 targeted the hsa-miR-5698. MTHFS and SHANK3 targeted the hsa-miR-4788.

## Discussion

In recent decades, there has been a consistent ascribing to a growing morbidity of LUAD, which is recognized as the most basic subtype of lung cancer. This increase has been attributed to an overall rise in the prevalence of lung cancer. Even though the vast majority of therapeutic methods, including as radiation, chemotherapy, targeted therapy, and surgical operations, have been employed in a variable manner to treat patients with LUAD, the OS rate has not been satisfactory. The growth, invasion, metastasis, and recurrence of LUAD are intricately tied to genetic alterations as well as immunological dysregulation in the humoral internal milieu (32). Because of the crucial part that the human immune system plays in the development of carcinoma (33), a variety of immunotherapeutic approaches have been developed to eradicate cancerous tumor cells (34). However, because persons who have been diagnosed with LUAD share a diversity of biological characteristics (35), different people have different reactions to actual therapeutic immunotherapy. This points to the possibility that some patients will have an unfavorable result from their treatment.

Low expression of METTL7A, a member of the human methyltransferase-like protein family (36–39), is associated with aggressiveness and progression of cancer malignancies tumor. In addition, Qi et al. (40) observed that a survival study of two individual hepatocellular carcinoma cohorts showed that downregulation of METTL7A in tumors predicts poor prognosis in HCC patients. The findings of the above studies confirmed the protective effect of METTL7A on the prognosis of HCC, and the same analytical results were found in LUAD. We speculated that METTL7A also has a similar effect in LUAD. Furthermore, they investigated the disruption of the interaction between adenosine deaminase acting on double-stranded RNA (dsRNA) and Dicer (41) or Drosha (42), which may represent a targetable method to rescue the expression of the tumor suppressor METTL7A, which could be used in the future as a novel approach for the treatment of HCC and other forms of cancer (40). Therefore, METTL7A was found to be a tumor suppressor in HCC. Felters et al. (39) using multiple bioinformatic approaches to analyze the collated omics data, they found that overexpression of METTL7A resulted in increased disease survival in patients with cutaneous melanoma. The results of this survey are also consistent with this analysis. METTL7A is a membrane protein that may be found on the inner omentum, which plays an important role in the innate immune system. There is also evidence that METTL7A is downregulated in thyroid cancer, suggesting that the cancer-specific DNA methylation signature of the METTL7A exon is important in tumor programming (36). On the other hand, the role of METTL7A in LUAD development has not been investigated. However, the results of the bioinformatic study gave us some indication that the gene METTL7A is a differentially expressed tumor suppressor throughout the development of LUAD. Therefore, based on our findings, we hypothesized that the reduction of METTL7A might contribute to the development of LUAD. However, this hypothesis must be supported by further studies.

According to this study, METTL7A was expressed at much lower levels in cancer tissues than in healthy tissues. overexpression of the METTL7A gene is an independent prognostic factor and is a good indicator of prognosis. LUAD samples were divided into high and low risk groups based on the median level of METTL7A expression. GSVA showed that a number of pathways associated with malignancy were substantially upregulated in the low-risk group. These included hypoxia (43–45), the p53 pathway (44, 46), KRAS signaling (47, 48), TGF signaling MTOR signaling pathways (49, 50), mitotic spindle (51), and other processes. All of the above data suggest that many pathways may be involved in the underlying mechanisms responsible for the poor prognosis of LUAD patients. Therefore, we raise the question that reduced expression of METTL7A may contribute to the activation of the above pathways and thus to the progression of LUAD. Furthermore, DNA repair is disproportionately strong in high-risk populations. According to scientific studies, DNA repair is essential for the body to maintain the structural integrity and stability of DNA, as well as to ensure the continuation of life and the stability of the species. There is a wide variety of mechanisms or processes in cells that are able to repair damaged DNA. Direct repair, excision repair, recombination repair, and trans-lesion repair are examples of common DNA repair pathways or systems (52). These forms of repair can prevent mutations in the cell, which can lead to cancer or even death. Nonetheless, it is intriguing to find that there are also pathways associated with tumour growth in high-risk patients. Some examples of these pathways include glycolysis (53, 54), WNT β-catenin signaling (55), and oxidative phosphorylation (55, 56). Based on the results of the GSVA study, there is evidence that downregulated METTL7A connects a number of related pathways and therefore promotes development and reduces prognosis of tumor patients. However, further testing is required to validate this idea.

On the other hand, it is important to highlight the function of genes and how they are associated with the development of cancer, which may provide important basic information that could help to improve the screening of candidate biomarkers (57). In particular, information about genes affecting the prognosis of cancer patients could provide insight into the molecular significance of candidate biomarkers for cancer being developed. YTHDC2 is now shown to inhibit LUAD by suppressing SLC7A11-dependent antioxidant activity (58), while METTL7A has good predictive power and clinical validity. However, little is currently known about the functional importance of biomarker genes in relation to cancer development. By combining data from TCGA-LUAD and GEO gene expression profiles, we were able to create a gene signature with predictive power. By using METTL7A, the TCGA-LUAD cohort could be divided into a low-risk group and a high-risk group and the data showed a significant difference in overall survival between the two groups. In addition, the discriminatory power of risk score, stage and METTL7A had significant advantages over models that included only each parameter alone. According to the results of the multivariate Cox regression study, the risk score was an independent predictor of prognosis in LUAD. In addition, the predictive accuracy of this prognostic feature was further assessed using nomograms plots and DCA. Predictive gene signatures have now been successfully developed by others for potential therapeutic targets for improving cancer patient prognosis (59–62). Taken together, these findings raise the possibility that one or more genes critical for predicting prognosis in cancer patients are refined potential biomarkers.

Although we found unique genetic features associated with OS, our study did have some limitations. The large number of cases lacked information including clinicopathological variables, and the sample size may have been insufficient; both factors may have contributed to the bias of the Cox regression survival analysis results. Although our findings are based on extensive bioinformatics studies, information including clinicopathological variables is lacking. In addition, further confirmation of METTL7A gene expression and predicted METTL7A gene activity in the Chinese cohort is required to properly interpret these findings. In addition, this study may be biased by gender, age, and treatment. Therefore, the predictive potential of the METTL7A gene must be validated in a large cohort with sufficient sample size and comprehensive clinicopathological features. Furthermore, to examine the biological importance of the METTL7A gene, further studies using animal models or cell lines will be performed to more thoroughly validate our findings.

## Conclusion

Through our study, we were able to identify METTL7A, which has the potential to serve as a predictive biomarker in LUAD. In addition, METTL7A, miRNAs, and lncRNAs were identified, and a ceRNA regulatory network was created to explain the underlying mechanism of METTL7A in LUAD. These findings may help to elucidate the molecular pathways involved in the initiation and progression of LUAD, thereby providing early diagnosis and new therapeutic targets for LUAD.

## Data availability statement

The original contributions presented in the study are included in the article/**Supplementary Material**. Further inquiries can be directed to the corresponding author.

## Ethics statement

The studies involving human participants were reviewed and approved by The Ethics Committee of the People’s Hospital Affiliated to Jiangsu University. The patients/participants provided their written informed consent to participate in this study. Written informed consent was obtained from the individual(s) for the publication of any potentially identifiable images or data included in this article.

## Author contributions

Y-QP conceived the manuscript. YX and Z-HL wrote the manuscript. LT, GC and J-FZ conducted the statistical analysis. LL, J-CL and J-QQ explain the results. A-ZS reviewed and edited the manuscript. All authors contributed to the article and approved the submitted version.

## Supplementary material

Supplementary Figure 1 Differences of survival based on clinical features. **(A)** Differences of survival between M0 and M1. **(B)** Differences of survival among tumor stage I, stage II, stage III and stage IV. **(C)** Differences of survival between age ≤65 and age > 65. **(D)** Differences of survival among N0, N1, N2 and N3 stage. **(E)** Differences of survival among T1, T2, T3 and T4 stage.
