# Supplementary figures and images for: Comprehensive analysis of the significance of METTL7A gene in the prognosis of lung adenocarcinoma
Source: Front Oncol. 2022 Dec 23;12:1071100. doi: 10.3389/fonc.2022.1071100 (PMC9817104; doi:10.3389/fonc.2022.1071100)

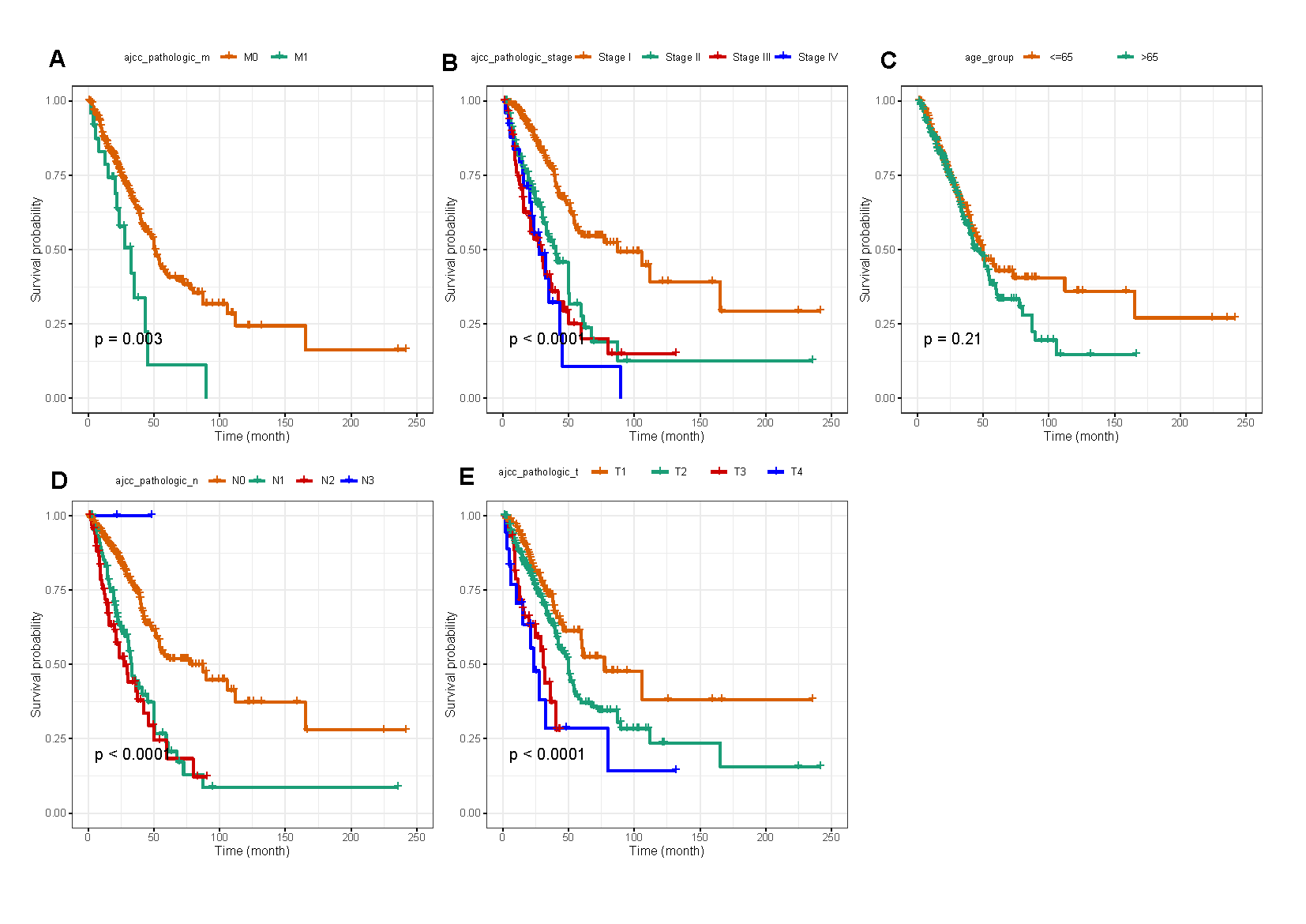

Supplement: Supplementary Figure 1 — Differences of survival based on clinical features. (A) Differences of survival between M0 and M1. (B) Differences of survival among tumor stage I, stage II, stage III and stage IV. (C) Differences of survival between age ≤65 and age > 65. (D) Differences of survival among N0, N1, N2 and N3 stage. (E) Differences of survival among T1, T2, T3 and T4 stage. [file Image_1.tiff]
